# Perioperative and long-term functional outcomes of robot-assisted versus open partial nephrectomy: A single-center retrospective study of a Japanese cohort

**DOI:** 10.1016/j.amsu.2022.103482

**Published:** 2022-03-08

**Authors:** Kiyoshi Takahara, Kosuke Fukaya, Takuhisa Nukaya, Masashi Takenaka, Kenji Zennami, Manabu Ichino, Hitomi Sasaki, Makoto Sumitomo, Ryoichi Shiroki

**Affiliations:** Department of Urology, Fujita Health University School of Medicine, Toyoake, Aichi, Japan

**Keywords:** Estimated glomerular filtration rate, Ischemia duration, Open partial nephrectomy, Robot-assisted partial nephrectomy, BMI, body mass index, EBL, estimated blood loss, eGFR, estimated glomerular filtration rates, LPN, laparoscopic partial nephrectomy, LRN, laparoscopic radical nephrectomy, OPN, open partial nephrectomy, ORN, open radical nephrectomy, PN, partial nephrectomy, RAPN, robot-assisted partial nephrectomy, RN, radical nephrectomy

## Abstract

**Objective:**

This study aimed to compare the perioperative and long-term functional outcomes between robot-assisted partial nephrectomy (RAPN) and open partial nephrectomy (OPN) in Japanese patients.

**Methods:**

We retrospectively analyzed 242 patients who underwent either RAPN or OPN between 2007 and 2017 at our hospital. Propensity score matching was carried out between the two groups at a ratio of 1:1. Perioperative outcomes and postoperative estimated glomerular filtration rates (eGFR) were compared at one and three years of follow-up.

**Results:**

After propensity score matching, we evaluated 39 patients from each group. The ischemia duration of the RAPN group was significantly shorter than that of the OPN group (18 vs. 24, *p* < 0.001). Moreover, the estimated blood loss (EBL) was significantly lower in the RAPN group than in the OPN group (50 vs. 174, *p* < 0.001). However, there were no significant differences in the postoperative eGFR between the two groups at one or three years of follow-up (OPN 54.8 vs. RAPN 61.2, *p* = 0.109, and OPN 54.8 vs. RAPN 55.5, *p* = 0.262, respectively).

**Conclusion:**

RAPN resulted in shorter ischemia durations and lower rates of EBL than did OPN; however, no differences in long-term renal function were observed between RAPN and OPN in our propensity-score matched Japanese cohort.

## Highlights

• This study aimed to compare the perioperative and long-term functional outcomes of RAPN and OPN in Japanese patients.
• RAPN resulted in shorter ischemia durations and lower rates of estimated blood loss than OPN.
• No differences in long-term renal function were observed between RAPN and OPN.

## 1 Introduction

Partial nephrectomy (PN) has been the standard treatment for small renal tumors for some time, as the long-term oncological outcomes and preservation of renal function rival those of radical nephrectomy (RN) [[1], [2], [3]]. In terms of the evolution of nephrectomy, open radical nephrectomy (ORN) has been replaced by open PN (OPN) which in turn, paved the way for minimally invasive PN, the latter of which includes both laparoscopic PN (LPN) and robot-assisted PN (RAPN) [4]. Laparoscopic RN (LRN) is a mature technique with a short learning curve that has been widely used by surgeons; moreover, LRN has been associated with fewer perioperative complications than its counterparts [5]. On the other hand, LPN is a challenging procedure with a steep learning curve. Hence, its adoption is limited among urologists [6,7]. Over the past decade, technological evolution and the adoption of robotic surgical platforms has spurred the popularity of RAPN. Large comparative studies and meta-analyses have previously reported the significant advantages of RAPN over LPN [[8], [9], [10]].

However, to date, comparisons of outcomes between RAPN and OPN in Japanese patients have not been widely introduced, especially regarding long-term functional outcomes. Therefore, in the present study, we aimed to compare the effects of RAPN on outcomes with those of OPN. The primary aim was to compare the perioperative outcomes of RAPN and OPN, and the secondary aim was to compare the long-term functional outcomes of them, using a propensity-score matched Japanese cohort.

## 2 Methods

### 2.1 Patient population

In this study, we retrospectively reviewed the clinical data of 242 patients who underwent either RAPN or OPN at Fujita Health University between August 2007 and September 2017, excluding those who had incomplete data, combined surgery, solitary kidney, multiple renal tumors, benign tumors, and metastatic tumors.

### 2.2 Surgery

We discussed the method of RAPN in our previous report [11]. Briefly, the tumor was resected with 2–5 mm of the parenchymal margin. For the inner renorrhaphy layer, the collecting system and large vessels were closed with 3–0 V-Loc sutures, and if needed, parenchymal sutures were made with 2–0 V-Loc. Seven surgeons who completed the da Vinci certification program approved in Japan performed RAPN.

During OPN, ice slush was placed around the kidney. The tumor was excised under cold, ischemic conditions involving clamping of the renal artery, and an adequate surrounding margin of normal renal tissue was retained. Opened calyces and bleeding sites were sutured, and the parenchymal defect was closed with horizontal, interrupted sutures.

### 2.3 Data collection

We recorded the following information preoperatively and at one and three years of follow-up: patient characteristics, including age, gender, and body mass index (BMI); clinical disease characteristics, including tumor side, RENAL score [12], and presence of a hilar tumor; surgical parameters, including operation time, ischemia duration, estimated blood loss (EBL, mL), need for blood transfusion, and presence of complications (Clavien-Dindo) [13]; and renal function findings, including estimated glomerular filtration rate (eGFR, mL/min/1.73 m^2^) as calculated using the Modification of Diet in Renal Disease equation [14]. All patients underwent preoperative cross-sectional imaging to evaluate location, tumor size, and depth of tumor to assess the RENAL score.

The protocol of this study was approved by the ethics committee of our institution (HM 16–340), and the study was performed in accordance with the ethical standards laid down in the most recent version of the Declaration of Helsinki. The need for informed consent from all patients included in this study was waived because of the retrospective design. The work has been reported in line with the STROCSS criteria [15].

### 2.4 Statistical analyses

Owing to inherent differences in baseline patient and disease characteristics between the RAPN and OPN groups, we used 1:1 propensity score-matched analysis to adjust for imbalances in confounding factors. The propensity scores of each patient were calculated using multivariable logistic regression. Comparisons between the groups were performed using either the chi-square test or Fisher's exact test. All data were analyzed using IBM SPSS Statistics version 23 (SPSS Japan Inc., Tokyo, Japan), and *p*-values of <0.05 were considered significant in all statistical analyses.

## 3 Results

### 3.1 Patients enrolled in the current study

**Fig. 1:**
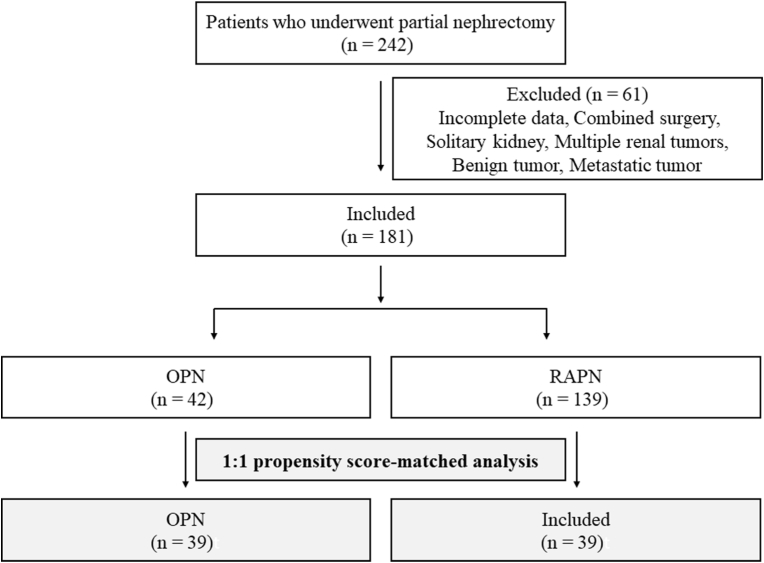
Flowchart of the patients enrolled in the study. We evaluated 242 patients who underwent partial nephrectomy. We included 181 patients in the present study and divided them into groups according to the operation method. Propensity score-matched analysis was performed thereafter.

In this study, we selected 242 patients who underwent PN at our hospital between August 2007 and September 2017. After excluding patients who did not fulfill our criteria, a total of 181 patients (OPN: 42, RAPN: 139) were enrolled. A 1:1 propensity score-matched analysis was performed, and 39 patients from each group were evaluated (Fig. 1).

### 3.2 Clinical characteristics of patients

Patient characteristics, including age, sex, BMI, tumor side, preoperative eGFR, RENAL score, and the presence of hilar tumors, were compared between the OPN and RAPN groups both before and after matching. In both cases, no significant differences in factors were observed between the OPN and RAPN groups (Table 1).

**Table 1:** Preoperative variables of pre- and post-matched patients.

	Pre-matching			Post-matching		
Median (range) or n (%)	OPN (n = 42)	RAPN (n = 139)	p value	OPN (n = 39)	RAPN (n = 39)	p value
Age	67 (35–78)	63 (29–88)	0.093	67 (35–78)	62 (29–85)	0.096
Gender (%)						
Male	32 (76.2)	111 (79.9)	0.666	29 (74.4)	27 (69.2)	0.802
Female	10 (23.8)	28 (20.1)		10 (25.6)	12 (30.8)	
BMI, kg/m^2^	24 (13–30)	24 (16–40)	0.847	24 (13–30)	23 (18–40)	0.721
Tumor side						
Right	17 (40.5)	70 (50.4)	0.293	15 (38.5)	18 (46.2)	0.647
Left	25 (59.5)	69 (49.6)		24 (61.5)	21 (53.8)	
Preoperative						
eGFR, ml/min/1.73m^2^	64 (18–101)	69 (31–136)	0.065	64 (27–101)	71 (31–103)	0.191
RENAL score	7 (4–11)	7 (4–10)	0.572	7 (4–11)	8 (4–9)	0.942
Hilar tumor	7 (16.7)	30 (21.6)	0.215	7 (17.9)	10 (25.6)	0.584

### 3.3 Perioperative outcomes

Following propensity matching, we compared the perioperative factors, including operation time, ischemia duration, EBL, need for transfusion, Clavien-Dindo classification ≥3, and postoperative eGFR at both one and three years of follow-up between the OPN and RAPN groups. Although there was no significant difference in the operation time between the two groups (OPN 169 vs. RAPN 175, *p* = 0.500), the ischemia duration was significantly shorter in the RAPN group than in the OPN group (18 vs. 24, *p* < 0.001). Moreover, the EBL was significantly lower in the RAPN group than in the OPN group (50 vs. 174, *p* < 0.001). No significant differences were observed between the OPN and RAPN groups in terms of transfusion and Clavien-Dindo classification (OPN 7.7% vs. RAPN 7.7%, *p* = 1.00, and OPN 5.1% vs. RAPN 5.1%, *p* = 1.00, respectively) (Table 2).

**Table 2:** Perioperative outcomes of post-matched patients.

	Post-matching		
Median (range) or n (%)	OPN (n = 39)	RAPN (n = 39)	p value
Operation time, min	169 (84–325)	175 (103–267)	0.500
Ischemia duration, min	24 (12–43)	18 (10–44)	<0.001
EBL, ml	174 (14–1083)	50 (5–1200)	<0.001
Transfusion	3 (7.7)	3 (7.7)	1.000
Clavien-Dindo ≧3	2 (5.1)	2 (5.1)	1.000

**Fig. 2:**
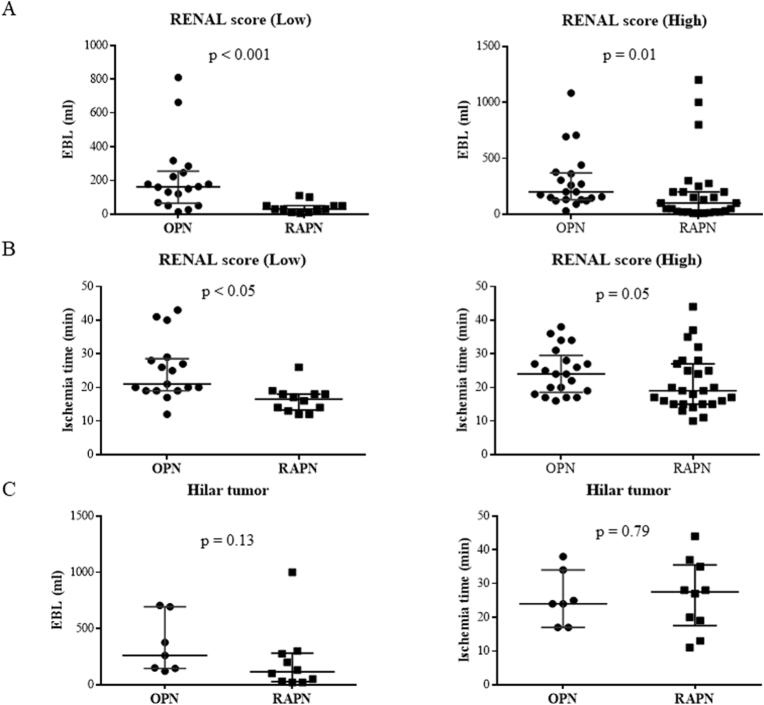
A. EBL of Low or High RENAL score (OPN vs RAPN) B. Ischemia duration of Low or High RENAL score (OPN vs RAPN) C. EBL and Ischemia duration of Hilar tumors (OPN vs RAPN).

We next assessed the two factors of ischemia duration and EBL in the RENAL score category. Each group was divided into the low group of RENAL score (4–6) or high group of RENAL score (7-12). The low RENAL score group included 30 patients (OPN: 18, RAPN: 12), while the high RENAL score group included 48 patients (OPN: 21, RAPN: 27). The EBL was significantly lower in the RAPN group than in the OPN group, both in the low and high groups of RENAL score (OPN 162 vs. RAPN 30, p < 0.001, and OPN 200 vs. 100, p = 0.01, respectively) (Fig. 2A), while the ischemia duration was significantly shorter in the low group of RENAL score (OPN: 21, RAPN: 16, p < 0.001), but not in the high group (OPN: 24, RAPN: 19, p = 0.05) (Fig. 2B). Regarding the presence of hilar tumors, 7 patients with OPN and 10 patients with RAPN had hilar tumors in the post-matching cohort. There were no significant differences between these two groups in either EBL or ischemia duration (OPN 260 vs. RAPN 115, p = 0.128, and OPN 24 vs. RAPN 28, p = 0.79, respectively) (Fig. 2C).

### 3.4 Long-term functional outcomes

**Fig. 3:**
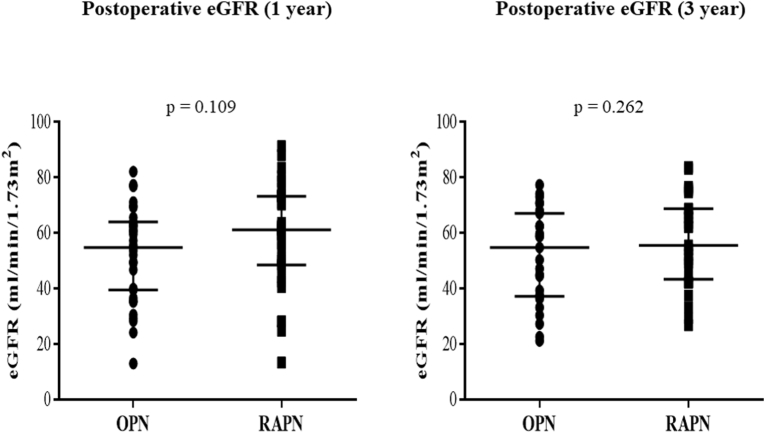
Postoperative eGFR at one or three years (OPN vs RAPN).

With respect to renal function, there were no significant differences in postoperative eGFR between the two groups at either one or three years of follow-up (OPN 54.8, RAPN 61.2, *p* = 0.109, and OPN 54.8 vs. RAPN 55.5, *p* = 0.262, respectively) (Fig. 3).

## 4 Discussion

Recently, several high-quality observational studies comparing RAPN and OPN have been published [[16], [17], [18], [19], [20], [21], [22], [23], [24], [25], [26], [27], [28]]. However, to date, the differences in the outcomes of RAPN and OPN especially in Japanese populations remain unknown. Therefore, in the current study, we focused on Japanese patients with renal tumors and compared the perioperative and long-term functional outcomes of RAPN and OPN.

Following 1:1 propensity score matching of the RAPN and OPN groups, 39 Japanese patients from each group were evaluated. Moreover, the ischemia duration of the RAPN group was significantly shorter than that of the OPN group (18 vs. 24, *p* < 0.001). The OPN procedure can be performed using the cold ischemia technique in order to prevent renal function, whereas RAPN is performed with the warm ischemia technique. Hence, it is unlikely that the difference in ischemia duration between RAPN and OPN affected renal function. Furthermore, Takagi et al. reported that patients undergoing RAPN had a shorter ischemia duration than those undergoing OPN (21 vs. 35 min, *p* < 0.001), as different ischemia methods were used (warm and cold ischemia in the RAPN and OPN groups, respectively) [24]. However, according to a recent systematic review and meta-analysis of comparative studies comparing perioperative outcomes between RAPN and OPN, the latter had a significantly shorter ischemia duration [29]. These controversial results ultimately depend on the tumor's anatomical complexity and the skill of the surgeon.

Regarding the EBL in the operation, it was significantly less in the RAPN group than in the OPN group (50 vs. 174, *p* < 0.001). As was the case in our study, Xia et al. demonstrated that the EBL of 2833 patients was significantly lower in patients undergoing RAPN than those undergoing OPN [29].

With respect to long-term renal function, we assessed the postoperative eGFR at one and three years after RAPN. Our study demonstrated that there were no significant differences in the postoperative eGFR at one or three years between the RAPN and OPN groups. This finding was supported by numerous recent studies with similar findings [17,[19], [20], [21],[23], [24], [25],[30], [31], [32]].

This study had some limitations. First, this was a retrospective, single-institution study. Second, in order to adjust for clinical and demographic imbalances, we performed a matched-pair analysis, which resulted in a small number of patients. Third, since several surgeons performed the RAPN and OPN operations, the technical proficiency of the operators may have varied.

In conclusion, RAPN and OPN showed similar long-term renal function outcomes in this Japanese cohort. However, a shorter ischemia duration and lower EBL was observed when RAPN was used.

### 4.1 Take-home messages

In the Japanese cohort, RAPN provides similar outcomes of long-term renal function to OPN, however, it appeared to be a valuable alternative to OPN, with the advantages of reduced ischemia time and blood loss.

## Authors’ contributions

The contributions of each author are as follows: Study conception and design, Kiyoshi Takahara, Ryoichi Shiroki; Acquisition of data, Kiyoshi Takahara, Kosuke Fukaya, Takuhisa Nukaya, Masashi Takenaka, Kenji Zennami; Analysis and interpretation of data, Kiyoshi Takahara, Manabu Ichino, Hitomi Sasaki, Makoto Sumitomo; Drafting of manuscript, Kiyoshi Takahara, Ryoichi Shiroki.

## Source of funding

No specific funding was received for the present analysis.

## Ethical approval

The protocol of this study was approved by the ethics committee of our institution (HM 16–340), and the study was performed in accordance with the ethical standards laid down in the most recent version of the Declaration of Helsinki.

## Registration of research studies

Name of the registry: Perioperative and long-term functional outcomes of robot-assisted versus open partial nephrectomy: a single-center retrospective study of a Japanese cohort
Unique Identifying number or registration ID: 7496
Hyperlink to your specific registration (must be publicly accessible and will be checked): performed

## Consent

The need for informed consent from all patients included in this study was waived because of the retrospective design.

## Provenance and peer review

Not commissioned, externally peer-reviewed.

## Guarantor

The Guarantor is Kiyoshi Takahara.

## Declaration of competing interest

The authors declare that there are no conflicts of interests.
